# Cancer and Non-Cancer Effects Following Ionizing Irradiation

**DOI:** 10.3390/cancers16061141

**Published:** 2024-03-13

**Authors:** Nobuyuki Hamada

**Affiliations:** Biology and Environmental Chemistry Division, Sustainable System Research Laboratory, Central Research Institute of Electric Power Industry (CRIEPI), Chiba 270-1194, Japan; hamada-n@criepi.denken.or.jp

## Abstract

On the one hand, ionizing radiation has been used to treat not only cancer, but also non-cancer diseases. On the other hand, associations with radiation exposure have increasingly been reported not only for cancer, but also non-cancer diseases, both at doses or dose rates much lower than previously suggested or considered. This underscores the need for considering both cancer and non-cancer effects of medical (diagnostic or therapeutic), occupational or environmental exposure to radiation. As such, this Special Issue aims to serve as a forum to gather the latest developments and discuss future prospects in the field of normal tissue responses to radiation exposure. The Special Issue is composed of 18 articles outlining the radiation effects arising in various tissues (e.g., those in the circulatory, sensory, nervous, respiratory, and reproductive systems).

## 1. Introduction

Ionizing radiation is used to treat cancer [1,2], but is also a carcinogen [3,4]. Alongside that, there has been mounting interest not only in radiotherapy for non-cancer diseases [5,6,7,8,9], but also the non-cancer effects of radiation exposure that occur at doses or dose rates much lower than previously suggested or considered [9,10,11]. This underlines the need to consider both the cancer and non-cancer effects of medical (diagnostic or therapeutic), occupational or environmental exposure to radiation. Therefore, this Special Issue (https://www.mdpi.com/journal/cancers/special_issues/cancer_ionizing_radiation) aims to serve as a forum to gather the latest developments and discuss future prospects in the field of normal tissue responses to radiation exposure. The Special Issue consists of 18 articles [12,13,14,15,16,17,18,19,20,21,22,23,24,25,26,27,28,29] outlining the cancer and non-cancer effects of radiation occurring in various tissues (e.g., those in the circulatory, sensory, nervous, respiratory, and reproductive systems), including studies on mitigation strategies and biomarkers, as outlined below.

## 2. Overview of Published Articles

### 2.1. Circulatory System

A growing body of epidemiological evidence has suggested elevated radiation risks of cardiovascular diseases (especially ischemic heart disease and stroke) [30,31,32,33]; however, the manifestations (in particular at low or moderate doses, and at low dose rates) and mechanistic underpinnings of this remain incompletely understood [9,34,35]. Nabialek-Trojanowska et al. [12] carried out speckle-tracking echocardiography in 12 patients at a median of 51 months after radiotherapy for mediastinal lymphoma, concluding that radiation exposure of the heart substructures is correlated with cardiac dysfunction (e.g., left ventricular global or anterior longitudinal strain). Honaryar et al. [13] conducted a prospective study of 101 breast cancer patients who received radiotherapy but not chemotherapy, and found that at two years after radiotherapy, early progression of calcification in the left anterior descending coronary artery is associated with radiation exposure of the left ventricle. Tanno et al. [14] performed microRNome analysis in the heart of wild-type mice whose whole bodies or partial (lower one-third) bodies were irradiated, and revealed the differential expression of microRNAs belonging to the myomiR family in the heart of whole body- or partial body-irradiated mice. Tanno et al. [14] also conducted in vitro experiments whereby irradiated skeletal muscle cells and non-irradiated ventricular cells were co-cultured, and proposed miR-1/133a as a potential mediator of the abscopal (out-of-field) response in non-directly irradiated tissues. Mpweme Bangando et al. [15] irradiated the aortic valves of mice defective in transient receptor potential melastatin 4 (TRPM4, monovalent non-selective cation channel) or their wild-type counterparts, and found that TRPM4 is involved in aortic valve remodeling after irradiation. Sridharan et al. [16] compared cardiac changes (e.g., plasma metabolomics, collagen deposition, mast cell numbers, and Toll-like receptor 4 expression) in wild-type mice whose whole hearts or partial (40%) hearts received irradiation, and observed no difference in adverse tissue remodeling in the irradiated and unirradiated parts of the heart. Azimzadeh et al. [17] conducted proteomic analysis in the heart of apolipoprotein E-deficient mice of which whole bodies were continuously exposed at 1 mGy/day or 20 mGy/day, and found that such chronic irradiation modulates various pathways in the heart that are common with age-related pathways. Hamada et al. [18] used four different irradiation regimens to deliver the same total dose, and found that the magnitude of damage arising at 12 months post-irradiation in the aorta of whole body-irradiated wild-type mice was greater in 25 fractions, smaller in 100 fractions, and much smaller in chronic exposure (at ca. 1 mGy/h) compared with acute, single exposure, confirming the results obtained at 6 months post-irradiation [36].

### 2.2. Sensory System

Regarding the effects of radiation exposure on the eye, evidence has accumulated for cataracts following moderate or high doses [37,38,39] (along with limited evidence at low doses [40,41]) and neovascular glaucoma following high doses [9]. Azizova et al. [19] reported a significantly increased radiation risk of normal-tension glaucoma (a subtype of primary open-angle glaucoma) in a cohort of Russian Mayak nuclear workers, confirming observations in Japanese atomic bomb survivors [42,43,44]. Thariat et al. [20] reviewed the current knowledge on normal tissue complications in the eye and orbit (e.g., the lacrimal gland, eyelashes, eyelids, cornea, lens, macula/retina, optic nerves and chiasma) following radiotherapy. Peuker et al. [21] found a sigmoidal relationship between radiation dose and the incidence of inner ear toxicity following radiotherapy for nasopharyngeal carcinoma, and proposed dose constraints to reduce inner ear toxicity.

### 2.3. Nervous System

Associations between radiation exposure and neurological effects on the brain have increasingly been reported [45,46,47,48,49]. Laurent et al. [22] conducted a cohort study of French nuclear workers and found significantly increased radiation risks of mortality from dementia and Alzheimer’s disease in addition to leukemia (excluding chronic lymphocytic leukemia), but not solid cancer. Rübe et al. [23] performed a survey of literature about the neurocognitive effects of radiation exposure and identified the age dependence of neurocognitive dysfunction following cranial radiotherapy, which was supported by pre-clinical rodent studies. Cantabella et al. [24] carried out transcriptomic analysis in the telencephalon of zebrafish exposed continuously at 0.05–5 mGy/h and found a dose rate-dependent increase in the genes involved in neurotransmission, neurohormones, and hypothalamic–pituitary–interrenal axis functions.

### 2.4. Respiratory System, Reproductive System, and Other Systems

Pertinent to the respiratory system, Matsuya et al. [25] examined the impact of local exposure to a radiocesium-bearing microparticle (an insoluble microparticle emitted by the incident at the Fukushima nuclear power plant [50,51]) in normal human lung fibroblasts and bronchial epithelial cells, and revealed the inflammatory signaling and DNA damage responses that were modified by the nuclear factor κB pathways. In relation to the reproductive system, Fukunaga et al. [26] reviewed current knowledge about radiation effects on spermatogenesis and its associated genotoxicity, and discussed the importance of preserving male fertility during radiotherapy from the perspective of oncofertility. Cruz-Garcia et al. [27] monitored the messenger RNA transcript abundance of DNA damage response genes in the circulating blood lymphocytes of patients with lung, neck, brain or pelvic cancer during radiotherapy, and found that ferredoxin reductase (FDXR) represents the most radioresponsive gene. In an effort to reduce radiation dermatitis following radiotherapy, Sörgel et al. [28] reported that hyaluronic acid and insulin-like growth factor I mitigated radiation-induced reductions in the viability and migration of human skin keratinocytes in vitro. Finally, Kuncman et al. [29] looked at the kinetics of FMS-related tyrosine kinase 3 ligand (Flt-3L, a multipotential hemopoietic factor) during chemoradiotherapy for rectal cancer and proposed the early initiation of immunotherapy when the concentration of Flt-3L is high and no lymphopenia has yet occurred.

## 3. Conclusions

I am grateful to the distinguished authors for their invaluable contributions and am indebted to the expert reviewers for their cooperation, dedication, and constructive comments. I would like to acknowledge *Cancers* for the opportunity to Guest-Edit this Special Issue. I hope that ongoing and future studies in this research field continue to give further insights into the manifestations and mechanisms of cancer and non-cancer effects following ionizing radiation exposure.
